# Augmenting BMI and Waist-Height Ratio for Establishing More Efficient Obesity Percentiles among School-going Children

**DOI:** 10.4103/0970-0218.51233

**Published:** 2009-04

**Authors:** Seeja Thomachan Panjikkaran, KS Kumari

**Affiliations:** Department of Food Science and Nutrition, Krishi Vigyan Kendra, Kerala Agricultural University, Tavanur, Kerala – 679 573, India

**Keywords:** BMI, children, India, methodology, nutrition, overweight, percentile chart, waist circumference, waist-height ratio

## Abstract

**Research Questions:**

1. Are all the existing methods for estimating the obesity and overweight in school going children in India equally efficient? 2. How to derive more efficient obesity percentiles to determine obesity and overweight status in school-going children aged 7-12 years old?

**Objectives:**

1. To investigate and analyze the prevalence rate of obesity and overweight children in India, using the established standards. 2. To compare the efficiency among the tools with the expected levels in the Indian population. 3. To establish and demonstrate the higher efficiency of the proposed percentile chart.

**Study Design:**

A cross-sectional study using a completely randomized design.

**Settings:**

Government, private-aided, unaided, and central schools in the Thrissur district of Kerala.

**Participants:**

A total of 1500 boys and 1500 girls aged 7-12 years old.

**Results:**

BMI percentiles, waist circumference percentiles, and waist to height ratio are the ruling methodologies in establishing the obese and overweight relations in school-going children. Each one suffers from the disadvantage of not considering either one or more of the obesity contributing factors in human growth dynamics, the major being waist circumference and weight. A new methodology for mitigating this defect through considering BMI and waist circumference simultaneously for establishing still efficient percentiles to arrive at obesity and overweight status is detailed here. Age-wise centiles for obesity and overweight status separately for boys and girls aged 7-12 years old were established. Comparative efficiency of this methodology over BMI had shown that this could mitigate the inability of BMI to consider waist circumference. Also, this had the advantage of considering body weight in obesity analysis, which is the major handicap in waist to height ratio. An analysis using a population of 1500 boys and 1500 girls has yielded 3.6% obese and 6.2% overweight samples, which is well within the accepted range for Indian school-going children.

**Conclusion:**

The percentiles for school-going children based on age and sex were derived by comparing all other accepted standards used for measurement of obesity and overweight status. Hence, augmenting BMI and waist to height ratio is considered to be the most reliable method for establishing obesity percentiles among school-going children.

## Introduction

Childhood obesity is alarmingly increasing worldwide(1) and it is linked with an increased risk of obesity in adulthood,(2) morbidity, and mortality.(3) Obesity has become so common that it is beginning to replace under nutrition and infectious diseases as the most significant contributor to ill-health. India is going through a nutrition transition phase and is now facing the double burden of nutrition disorders. Poor rural and urban slum populations have a high prevalence of under nutrition and on the other side, the newly rich urban, middle, and high income populations suffer from an emerging problem of obesity due to changing lifestyles and diet.(4)

The increased prevalence of pediatric obesity and its associated morbidities demonstrates the need for a simple anthropometric tool that can be used to assess and identify children who are at risk of becoming obese and subsequently require appropriate intervention. Because of their public health importance, the trends in child obesity should be closely monitored. Trends are, however, difficult to quantify and compare as a wide variety of definitions of child obesity are in use and no commonly accepted standards have yet emerged. BMI centile curves,(5) waist circumference centiles,(6) and waist to height ratio(7) are some of the accepted standard measures to determine obesity among children. A workshop organised by the International Obesity Task Force proposed that adult cut off points be linked with body mass centiles for children to provide child cut off points.(8) BMI centile curves have been developed for use in the pediatric population for clinical and possibly epidemiological purposes.(5) It has been suggested however, that BMI may be a less sensitive indicator of obesity among children, since it gives no indication about fat distribution. During growth in childhood, body fat is laid down both subcutaneously and intra abdominally, hence obtaining information on waist circumference in children could be as useful as BMI as a means of identifying the overweight and obese status in childhood population studies.(6) Waist circumference as a measure of obesity and overweight status suffers from the disadvantage of not considering important criteria such as body weight and height. A waist-to-height ratio can also be used as an indicator for obesity. A waist-to-height ratio (W/Ht) has been reported to be an effective predictor of metabolic risks in all related investigations, which may be due to better measurement of the relative fat distribution among subjects of different ages and statures and the possible independent effect of height on the metabolic risks in addition to its independent effect on coronary disease itself. A waist-to-height ratio has also been reported to have closer values between men and women than BMI or waist circumference; therefore, the same boundary value may apply to both men and women. Meanwhile, a waist-to-height ratio of 0.5 may be a simple and effective index not only to identify almost overweight children, but also to identify children within the normal weight range.(7) Even then, a perfect relation of this factor with obesity or overweight status is yet to be standardized and efforts in this line have only shown that individuals with scores above 0.5 are likely to fall in the category of either overweight or obese, irrespective of age.(9)

At this juncture, a new methodology is being prepared that considers all the growth dynamics by combining BMI with waist-to-height ratio for establishing standard obesity percentiles among school-going children. Details on the prevalence of obesity and overweight status among school-going children in India were gathered using the established standards. These results were compared to assess their relative efficiencies in relation to the expected levels in the Indian population and to demonstrate the higher efficiency of the proposed percentile chart, which were the major objectives of this study.

## Materials and Methods

The study on “Augmenting BMI and waist-height ratio for establishing an efficient obesity percentile among Indian school going children” was carried out to determine the prevalence and to establish a standard definition for obesity among school-going children (7-12 years old).

### Selection of samples

A cross-sectional, completely randomized design in multi stages was the sampling design adopted for this study. Various schools (Government, private aided, unaided, and central) in the Thrissur district of Kerala were the first stage units. Out of the total number of schools, 16 schools were randomly selected for this study. The second stage consisted of school children between the age group of 7-12 years old. The sample size was 16 at the first stage and in the second stage the total sample size was fixed at 3000 consisting of 1500 boys and 1500 girls each. The number of children selected from each school was in proportion to the total number of children in the school.

In this study, anthropometric measurements of 3000 school-going children were recorded using standardized procedures. Weights of children were recorded using a bathroom scale, which was checked by calibration with standard weights.(10) For measuring height, the subject was made to stand erect looking straight on a level surface with heels together and toes apart, without shoes. Height was read to the nearest of 0.5 cm. An average of three measurements were taken as the final measurement.(10) Waist circumference was measured midway between the lower rib margin and the iliac crest with a plastic tape to the nearest 1 mm. A non-elastic flexible tape was employed to measure the waist circumference with the subject in the standing position.(11,12) Waist circumference was compared with waist percentile charts given by McCarthy, *et al*.(13) to determine the prevalence of obesity.

Body Mass Index was derived using the following equation: weight (kg) / height (M)^2^ and the results were compared with percentile charts(5) to identify obese and overweight children. The waist-to-height ratio was calculated and compared with the standards.(9) The height and waist circumference of children increases continually as they age, the same boundary value (WHTR=0.5) could be used to indicate increased risk across all age groups.(13)

Based on the percent population identified in each methodology and by comparing the level of each sample obtained from all other measures, the centile values for each age was delineated separately for boys and girls, so that a balance in measurement was brought among all other methods. Using a BMI × waist-to-height ratio factor, the relative efficiency with respect to the capability of the model to pick out the exactly obese or over-weight samples from the population under this cross-sectional study was also tested.

## Results

**Figure 1:**
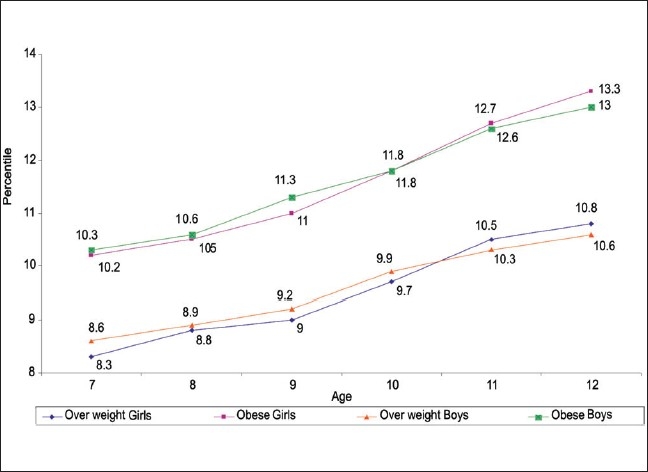
Age-wise obesity percentiles (BMI × Waist-Height ratio) for school going boys and girls aged 7-12

Based on the augmented values of the BMI and waist-to-height ratio, minimal percentile values for assessing the obesity and overweight status for Indian school children aged between 7 to 12 years were plotted [Figure 1]. The percentiles for school-going children based on age and sex were derived by comparing all other accepted standards used for measurement of obesity and overweight status. The individuals who were obese and overweight using all the other methodologies were used to define the minimal percentile values for defining obesity and overweight status in this present methodology.

The prevalence of obesity using various standardized procedures in the sampling population is presented in Table 1. Only 3.2% of the children were found to be obese using BMI percentiles based methodology whereas 8% of the children were found to be overweight. Measurement of waist circumference showed a four-fold increase in obese children; school-aged children who were first classified as overweight were considered to be obese. A comparison between waist circumference and BMI showed that at least 53.2% of the children who were obese using waist circumference were either overweight or normal using BMI. The waist-to-height ratio reveals that 16.8% of the samples were at risk. A child's height reveals the past nutritional status and is closely related to genetic factors. Weight is an indicator of present nutritional status and is influenced by environmental factors, hence, while considering obesity, it is necessary to consider all these factors to attain a standard definition for obesity. Augmenting BMI and waist-to-height ratio is more accurate with a prevalence of 3.6% and 6.2% of obese and overweight school children, respectively. These observed prevalence rates are well within the accepted limits of Indian school children.(12)

**Table 1:** Comparative efficiency of various methods for determining the overweight and obesity status in school-going children

	BMI		Waist circumference		Waist-height ratio under risk	BMI × Waist-height	
				
	Obese	Overweight	Obese	Overweight		Obese	Overweight
Percentage	3.2	8	13	4.2	16.8	3.6	6.2
Number out of 3000	96	240	390	126	504	108	186

## Discussion

The percentile chart developed by augmenting BMI and waist-to-height ratio depicted in Figure 1 is found to be more efficient in classifying obese and overweight school-going children as the samples that showed obese and overweight status using all the methodologies were used to define the percentiles for defining obesity and overweight status in this present methodology. The percentiles were derived considering all standard modes used for classifying obese and overweight school children. The prevalence of obesity using various standardized procedures in the sampling population is presented in Table 1. Only 3.2 and 8% of the children were found to be obese and overweight, respectively using BMI percentiles based methodology.(5) Ramachandran, *et al.*(14) studied children from six schools in Chennai, two each from high, middle, and lower income groups. The prevalence of overweight (including obese) adolescents ranged from 22% in better off schools to 4.5% in lower income group schools. In a Delhi school with tution fees more than Rs. 2,500 per month, the prevalence of overweight children was 31%, of which 7.5% were frankly obese.(15)

The arguments by McCarthy, *et al*.(6) suggests that BMI may be a less sensitive indicator of fatness among children and give no indication about fat distribution, which was again supported in our experiments. Many Asian races show a tendency for fat deposition in the abdominal area, which is known as central adiposity. Waist circumference is recommended as an index for central fat distribution but there is no global standard for it.(16)

A comparison between waist circumference and BMI had shown that at least 53.2% of the children who were obese using waist circumference were either overweight or normal using BMI. During growth in childhood, body fat is laid down both subcutaneously and intra-abdominally. The relationship between an increasing waist circumferences in obese children 12 to 14 years old with an adverse lipoprotein profile has been observed.(17) Secondly, data from the Bolgousa Heart study showed that an abdominal fat distribution (indicated by waist circumference) in children between 5 and 17 years old was associated with an adverse concentration of triacyl glycerol, LDL cholesterol, HDL cholesterol, and insulin.(18) Moreover, it is a straightforward methodology for predicting cardio-vascular disorders and could be adopted as an alternative or additional measurement to BMI in children(6) and correlates well with BMI in comparison to waist hip ratios in adults.(19) However, a waist circumference based analysis was proved to be over sensitive with 13% of the population estimated as obese against 4.2% who are overweight. Studies point out that the prevalence of child obesity in the Indian population is below 4%(20) and 13% against the observed value of 3.2% using BMI percentiles is less acceptable.

The waist-to-height ratio is more sensitive than BMI as an early warning of health risks. It is significantly associated with all risk factors for obesity and metabolic syndrome and can predict morbidity and mortality in longitudinal studies, often better than BMI.(9) Waist-to-height ratio is a simple and effective global indicator for health risks(21,22) but the risk lies in over sensitivity of this methodology as proven from the present observations. Since the height and waist circumference of children increases continually as they age, the same boundary value (WHTR-/0.5) could not be used across all age groups.(13) However, our study had shown that when using a waist-to-height ratio, as much as 16.8% of the population was at risk. This result suffers from the drawback that it is a very high prevalence rate compared with that from the BMI percentile methodology (11.2%) and the cut off value was observed to be less than that standardized (0.5) value. Our experiments had shown the cut off value to be 0.45. Further, this methodology fails to differentiate the obese population from the overweight population. In the waist-to-height ratio methodology, there is neither consideration for body weight nor age-wise recommendation for children. Hence, the percentile values derived through combining BMI and waist-to-height ratio are more reliable for determining obesity and overweight status in school children.

## Conclusion

A percentile chart for Indian school-going children derived as a product of BMI and waist-to-height ratio is presented. Using this, 3.6% of the population was found to be obese and 6.2% were found to be overweight. This observed prevalence is well within the normally reported rate in India.(23) The aberrations leading to inaccurate conclusions in other methodologies due to their inability to consider one or more of the essential parameters or over sensitivity are mitigated here. BMI merely takes into account weight and height, ignoring an equally important factor, waist circumference. The waist-to-height ratio considers waist circumference and height only, neglecting weight, the major indicator of obesity. The percentiles for school-going children based on age and sex were derived by a comparison with all other accepted standards used for measurement of obesity and overweight status, and the superiority of the same over conventional methodologies is demonstrated.
